# Long-Term Functional Outcomes and Correlation with Regional Brain Connectivity by MRI Diffusion Tractography Metrics in a Near-Term Rabbit Model of Intrauterine Growth Restriction

**DOI:** 10.1371/journal.pone.0076453

**Published:** 2013-10-15

**Authors:** Miriam Illa, Elisenda Eixarch, Dafnis Batalle, Ariadna Arbat-Plana, Emma Muñoz-Moreno, Francesc Figueras, Eduard Gratacos

**Affiliations:** 1 Department of Maternal-Fetal Medicine, Institut Clinic de Ginecologia, Obstetricia i Neonatologia (ICGON), Hospital Clinic, Barcelona, Spain; 2 Fetal and Perinatal Medicine Research Group, Institut d'Investigacions Biomèdiques August Pi i Sunyer (IDIBAPS), University of Barcelona, Barcelona, Spain; 3 Centro de Investigación Biomédica en Red de Enfermedades Raras (CIBERER), Barcelona, Spain; Université de Montréal, Canada

## Abstract

**Background:**

Intrauterine growth restriction (IUGR) affects 5–10% of all newborns and is associated with increased risk of memory, attention and anxiety problems in late childhood and adolescence. The neurostructural correlates of long-term abnormal neurodevelopment associated with IUGR are unknown. Thus, the aim of this study was to provide a comprehensive description of the long-term functional and neurostructural correlates of abnormal neurodevelopment associated with IUGR in a near-term rabbit model (delivered at 30 days of gestation) and evaluate the development of quantitative imaging biomarkers of abnormal neurodevelopment based on diffusion magnetic resonance imaging (MRI) parameters and connectivity.

**Methodology:**

At +70 postnatal days, 10 cases and 11 controls were functionally evaluated with the Open Field Behavioral Test which evaluates anxiety and attention and the Object Recognition Task that evaluates short-term memory and attention. Subsequently, brains were collected, fixed and a high resolution MRI was performed. Differences in diffusion parameters were analyzed by means of voxel-based and connectivity analysis measuring the number of fibers reconstructed within anxiety, attention and short-term memory networks over the total fibers.

**Principal Findings:**

The results of the neurobehavioral and cognitive assessment showed a significant higher degree of anxiety, attention and memory problems in cases compared to controls in most of the variables explored. Voxel-based analysis (VBA) revealed significant differences between groups in multiple brain regions mainly in grey matter structures, whereas connectivity analysis demonstrated lower ratios of fibers within the networks in cases, reaching the statistical significance only in the left hemisphere for both networks. Finally, VBA and connectivity results were also correlated with functional outcome.

**Conclusions:**

The rabbit model used reproduced long-term functional impairments and their neurostructural correlates of abnormal neurodevelopment associated with IUGR. The description of the pattern of microstructural changes underlying functional defects may help to develop biomarkers based in diffusion MRI and connectivity analysis.

## Introduction

Intrauterine Growth Restriction (IUGR) due to placental insufficiency occurs in 5–10% of all gestations [1] and it is thought to increase due to the delay in the maternal childbearing in modern societies [2]. Chronic reduction of placental blood supply results in sustained exposure to hypoxemia and undernutrition with the subsequent consequences on the developing brain [3]. The association between IUGR and short- term neurodevelopmental dysfunctions has been extensively described [4]–[9]. During neonatal period, term IUGR have poorer neurobehavioral and cognitive performance when compared with control term infants [4]. These neurobehavioral impairments seem to be even more pronounced in preterm IUGR when compared with term IUGR newborns, although these differences were not observed at long-term period [5]. Besides this, long-term follow-up studies have reported that abnormal neurodevelopment after IUGR persists until late childhood and adolescence [3], [10]–[24]. Recent reports have shown that children born with IUGR have long-term cognitive impairment and learning difficulties in school [13], [14], [17], [20], [22], being related to a characteristic pattern involving short-term memory, attention and anxiety, and increased risk of attention-deficit hyperactivity disorder (ADHD) [13]–[15]. These abnormalities have been suggested to reflect changes in specific areas including the anterior hippocampal-prefrontal network, parahippocampal complex, striatum, and thalamus [13], [14], [25]–[27]. Magnetic resonance imaging (MRI) studies have consistently demonstrated structural brain changes in IUGR during fetal and neonatal period including changes in brain texture analysis [6] and decreased volume in cortical grey matter (GM) [7], in the hippocampus [8] and differences in cortical development [9]. However, no long-term imaging studies have evaluated the neurostructural substrates underlying functional impairments in IUGR. This knowledge is required to explore the development of imaging biomarkers for early diagnosis and monitoring of abnormal neurodevelopment of prenatal origin [28].

IUGR is associated with disruption of normal brain neurodevelopment rather than gross tissue destruction [29], requiring the use of MRI modalities to identify subtle structural changes. Diffusion MRI is a noninvasive approach based on the measurement of the diffusion of water molecules in tissues [30], which provides indirect information about brain microstructure. Diffusion MRI has been used to assess brain reorganization in response to brain injury in both developing and adult brain [31], [32]. Specifically, diffusion MRI has been shown to detect changes occurring in IUGR [33]–[35] and other fetal conditions also associated with reduced brain oxygen supply such as fetal cardiac defects [36]. Aside from water diffusion parameters, quantitative tractography metrics can be obtained in order to estimate the connectivity of WM pathways among brain regions regulating specific brain functions. This approach has been used to identify changes in diseases of neurodevelopment such as ADHD [37], autism spectrum disorders [38] and periventricular leucomalacia [39], [40].

Evaluation of the long-term effects of IUGR on the human brain is limited by the difficulty of conducting prospective studies in sufficiently large sample sizes, and the potential influence of uncontrolled environmental factors [41]. Notwithstanding the obvious limitations, animal models provide the opportunity to conduct comprehensive studies spanning long maturational periods in homogeneous groups. Aside from the reproducibility of experimental conditions, MRI can be performed in isolated whole brain preparations allowing very long acquisition times with high-resolution [42]. The rabbit is a suitable model to reproduce IUGR [43]–[46] and it presents a human-like timing of perinatal brain WM maturation [44]. We have previously used this model to describe regional changes in fractional anisotropy in newborns which correlated with poorer outcome in neurobehavioral tests [34].

In this study we aimed at providing a comprehensive description of the long-term functional and neurostructural correlates of abnormal neurodevelopment associated with IUGR using a near-term rabbit model. Furthermore, we evaluated the development of quantitative imaging biomarkers of abnormal neurodevelopment based on regional changes in diffusion MRI parameters and connectivity. For all these purposes, we firstly assessed long-term neurodevelopment at a preadolescent equivalent age with functional tests extensively used in rodents. We tested the hypothesis that the rabbit model would display similar changes to humans, involving short-term memory, attention and anxiety problems. Secondly, brain microstructural changes were studied by means of diffusion MRI with high angular resolution schemes. Differences in diffusion parameters were analyzed by voxel-based analysis (VBA), to avoid the need for a priori hypothesis or previous delineation [47]. We also evaluated the presence of differences in the connectivity between brain areas described to be involved in anxiety and attention (including amygdala, hippocampus formation, striatum, thalamus and prefrontal, temporal and cingulate cortices) and short-term memory (including hippocampal formation, hippocampus, thalamus, prefrontal and temporal cortices). Finally, VBA and connectivity results were also correlated with the functional outcomes.

## Materials and Methods

**Figure 1:**
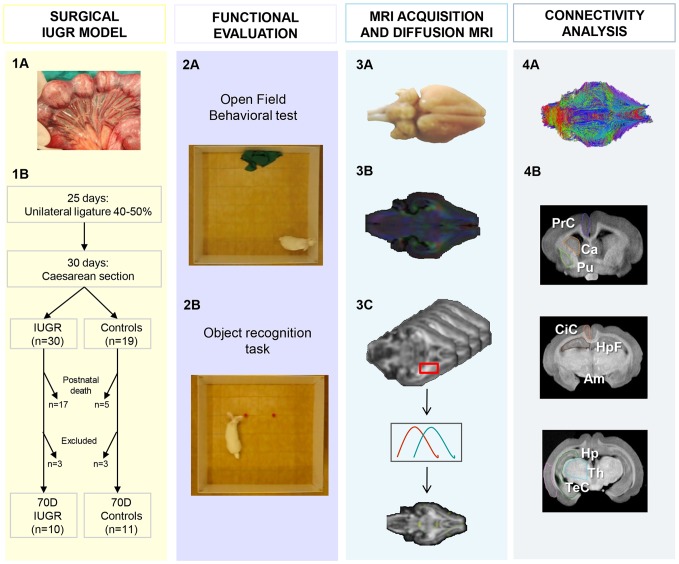
Schematic and graphical representation of the study design and methods. **PANEL 1**: (A) Illustrative image of unilateral ligation of 40–50% of uteroplacental vessels at 25 days of pregnancy, (B) Scheme of surgical procedures and study groups. **PANEL 2**: Illustrative pictures of neurobehavioral and cognitive evaluation in the Open Field Behavioral Test (A) and Object Recognition Task (B). **PANEL 3**: MRI acquisitions: (A) Fixed brains were scanned to obtain high resolution T1 weighted images and diffusion-weighted images. After masking brain volume, (B) global analysis was performed to obtain average DTI parameters (FA, linearity, planarity and sphericity coefficients). (C) Then voxel-based analysis of diffusion-related parameters was performed by elastic registration to a reference FA map. **PANEL 4**: (A) Illustrative image of tractography used for connectivity analysis. It was performed by measuring the ratio of fibers involved in anxiety and short-term memory networks over the total number of fibers reconstructed. (B) Manual delineation of brain regions involved in anxiety, attention and memory networks in coronal slices including prefrontal cortex (PrC), striatum (Ca + Pu), cingulate cortex (CiC), temporal cortex (TeC), thalamus (Th), amygdala (Am), hippocampus (Hp) and hippocampus formation (HpF).

The methodology of the study is shown in Figure 1. Each step of the procedure is detailed in this section.

### 1- Study protocol and procedures

#### 1.1- Ethics Statement

The animal experimentation of this study was approved by the Animal Experimental Ethics Committee of the University of Barcelona (permit number: 206/10–5440). Animal handling and all the procedures were performed following all applicable regulations and guidelines of the Animal Experimental Ethics Committee of the University of Barcelona, and all efforts were made to minimize suffering.

#### 1.2- Animals and study protocol

The study groups were composed of 10 cases with induced IUGR and 11 sham controls obtained from New Zealand pregnant rabbits provided by a certified breeder. Dams were housed for 1 week before surgery in separate cages on a reversed 12/12 h light cycle, with free access to water and standard chow. At 25 days of gestation (term at 31 days), we performed ligation of 40–50% of uteroplacental vessels following a previously described protocol [45] in 10 pregnant rabbits. Cesarean section was performed at 30 days of gestation and living pups were obtained. On the 70th postnatal day, which is considered to be equivalent to preadolescence period in humans in terms of sexual maturity [48], functional tests were applied and the rabbits were sacrificed thereafter. The brains were then collected and fixed with 4% paraformaldehyde phosphate-buffered saline (PBS).

#### 1.3- Surgical model

Briefly, after midline abdominal laparotomy, the gestational sacs of both horns were counted and numbered. Afterwards, only one uterine horn was kept outside the abdomen and the induction of IUGR proper was performed by ligating 40–50% of the uteroplacental vessels of all the gestational sacs from this horn. After the procedure, the abdomen was closed in two layers and postoperative analgesia (meloxicam) was administered for 48 hours. After surgery, the animals were allowed free access to water and standard chow for 5 days until delivery. Cesarean section was performed at 30 days of gestation and living and stillborn fetuses were obtained. All living newborns were weighed and identified by a subcutaneous microchip inserted in their back (Microchip MUSICC, Avid Microchip S.L., Barcelona, Spain). Cases were considered those pups delivered from the ligated horn, whereas controls were those delivered from the contralateral horn (non-ligated). Both cases and controls were housed with a wet nurse rabbit with part of the offspring (total number of rabbit pups in all litters: 8) until the 30th postnatal day when they were weaned. Thereafter both groups of rabbits were housed in groups of three with a reversed 12/12 h light cycle with free access to water and standard chow.

#### 1.4- Neurobehavioral and cognitive evaluation: functional tests

In order to assess functional changes, especially those related to emotion and cognition, two standard tests used extensively in rodents, such as the Open Field Behavioral Test and Object Recognition Task, were adapted for application in rabbits. Specifically, the Open Field Test evaluates locomotion and exploratory activities that compete against fear, anxiety and attention [49]–[51]. The Object Recognition Task evaluates declarative short-term memory, specifically recognition [52], as well as attention capacity [53] and is based on the tendency of rodents to explore new stimuli for a longer time compared to familiar stimuli [54]–[56]. Both tests were performed by placing each animal in a squared arena (140 cm ×140 cm) surrounded by opaque plastic walls (height 40 cm). First, we evaluated the Open Field Test with their first contact to the novel environment. As we sought to evaluate any degree of anxiety, we decided not to habituate the animals to the novel area as suggested previously [57]. After the Open Field Test, the animals were removed from the arena and in 30 to 60 minutes were again placed in the arena to evaluate the Object Recognition Task. Both tests were applied between 10 am to 5 pm and after each session the exploring area was cleaned with a 10% ethanol in order to erase any olfactory cue. The room was insulated from sound and with full overhead illumination. To minimize interference due to human contact, each session was video-taped and later evaluated by two blinded observers (MI, AAP).

The Open Field Behavioral Test was designed and used in accordance with the procedure previously described [51]. The testing area was divided into 36 squares of 23×23 cm, the 4 central squares were considered as the internal area and the remaining squares were defined as the peripheral area. For testing, the rabbits were taken out of their cage wrapped with a cloth and placed close to one of the lateral walls (starting point) and behavior was assessed during 10 minutes. Multiple parameters were recorded including latency of leaving the starting point (seconds), number of squares explored (internal or external), total time spent in internal and peripheral areas (seconds) and other general activities such as number of rearing and grooming.

The Object Recognition Task was performed, being adapted from the original description [58] including some modifications in the stimulus used. Instead of using visual stimulus, odour-based stimulus was used by means of placing pieces of fruit (apple or orange) inside perforated plastic boxes, since olfactory sensitivity is highly developed in rabbits [59]. This is in agreement with the notion that the type of stimulus presented must be one in which the sensory perception of the species chosen is adequate [59]. The test was divided into two consecutives phases. First, two boxes containing the same odour-based stimuli (apple) were presented to the animal during 5 minutes. This constituted the Familiarization phase. The rabbit was then returned to its cage for a 30-minute retention interval. Then, one of the objects was removed and replaced by a novel stimulus (orange) and the animal was again placed in the area with the novel and familiar objects for 5 minutes more in the Testing phase. Exploration of the object was considered when the rabbit showed sniffing, touching and having moving vibrissae while directing the nose towards the object at a distance of less than 1 cm. Cumulative time (seconds) exploring each object in the two sessions was recorded (right and left objects in the Familiarization phase, whereas novel and the familiar objects in the Testing phase). Finally, the discrimination index (DI), which represents the ability to discriminate the novel from the familiar object, was calculated as follows: DI =  (Novel Object Exploration Time – Familiar Object Exploration Time)/(Novel Object Exploration Time + Familiar Object Exploration Time). Learning criteria was considered when the DI was above 0. Animals that did not explore the familiar object at least once in the Testing phase or did not explore any of the objects in the Familiarization phase were excluded from the analysis, as previously suggested [60].

#### 1.5- Sample collection

After the functional tests, the rabbits were anaesthetized with ketamine 35 mg/kg and xylazine 5 mg/kg given intramuscularly and were sacrificed with an endovenous overdose of sodium pentobarbital (200 mg/kg). The left and right common carotid arteries were cannulated and the brains were perfused with phosphate-buffered saline (PBS) followed by 4% paraformaldehyde PBS. Finally, the brains were dissected and fixed in 4% paraformaldehyde PBS at 4°C for 48 h.

#### 1.6- Magnetic resonance acquisition

MRI was performed on fixed brains using a 7T animal MRI scanner (BrukerBioSpin MRI GMBH). First, high-resolution three-dimensional T1 weighted images were obtained in the brain samples by a Modified Driven Equilibrium Fourier Transform (MDEFT) 3D sequence with the following parameters: Time of Echo (TE)  = 3.5 ms, Time of Repetition (TR)  = 4000 ms, 0.7 mm slice thickness with no interslice gap, 70 coronal slices, in-plane acquisition matrix of 184×188 and Field of View (FoV) of 28×28 mm^2^, resulting in a voxel dimension of 0.15×0.15×0.7 mm^3^. Any potential tissue alteration, mainly significant tissue loss that could alter the results of further image-based analysis, was considered as exclusion criteria. Afterwards, diffusion-weighted images (DWI) were acquired using a standard diffusion sequence covering 126 gradient directions with a b-value of 3000 s/mm^2^ together with a reference (b = 0) image. Other experimental parameters were: TE = 26 ms, TR = 250 ms, slice thickness  = 0.7 mm with no interslice gap, 70 coronal slices, in-plane acquisition matrix of 40×40 , FoV of 28×28 mm^2^, resulting in a voxel dimension of 0.7×0.7×0.7 mm^3^. The total scan time for both acquisitions was 13 h56 m40 s.

### 2- MRI processing and analysis

#### 2.1- Post-processing MRI

As a first step, the brain was segmented from the background by means of customized software implemented in Matlab 2011a (The MathworksInc, Natick, MA, USA) similar to what has been described previously [34].

Tensor model of diffusion MRI was estimated at each voxel inside the brain mask [61] using MedINRIA 1.9 [62] (Inria Sophia Antipolis website, available at www-sop.inria.fr/asclepios/software/MedINRIA/. Accessed 2013 September 1). Based on the tensor model, a set of measures describing the diffusion were computed: fractional anisotropy (FA) and the coefficients of linearity, planarity and sphericity [30], [63]. These are all based on the three eigenvalues of each voxel tensor (λ1, λ2, λ3). FA describes the anisotropy of the diffusion, being higher in areas occupied by WM tracts [30]. Linearity, planarity and sphericity coefficients describe the shape of the diffusion. High values of linearity indicate that diffusion occurs mainly in one direction, which mainly involves the presence of fiber tracts; high planarity indicates that diffusion is performed mostly in one plane, which could be related to crossing fibers; and high values of sphericity are related to isotropic diffusion [63]. In addition, the orientation diffusion function (ODF) of each voxel was also reconstructed following a Q-Ball approach [64]. The ODF of each voxel was used to reconstruct fiber tracts by means of the deterministic tractography algorithm implemented in MedINRIA 1.9 [62] (Inria Sophia Antipolis website, available at www-sop.inria.fr/asclepios/software/MedINRIA/. Accessed 2013 September 1).

#### 2.2- Voxel-based analysis

To identify regional changes in diffusion-related parameters, VBA was performed. This analysis consists of the normalization of all the volumes to a reference volume and the comparison of the values at the same voxel of all the normalized volumes, thus identifying statistically significant differences. Registration of the diffusion tensor imaging (DTI) volumes to the reference was performed by means of a block matching algorithm, based on a DTI-specific metric [65]. Moreover, to preserve the coherence between DTI orientation information and the transformed volumes, the Preservation of Principal Direction (PPD) algorithm was applied [66]. In order to compensate for possible misregistrations and reduce noise effects, the registered volumes were smoothed. This smoothing also reduces the effective number of multiple comparisons in the statistical testing, thereby improving statistical power [67]. Van Hecke et al. [68] stated that anisotropic smoothing leads to more accurate VBA results, since it preserves the edges between different kinds of tissues, reducing the partial volume effects. For this reason, we applied an anisotropic Wiener filter [69] to the registered volumes. Once the images are aligned to the reference, it can be assumed that voxels in the same location in all the registered images belong to the same structure, and therefore, they can be compared. Voxel-wise t-test was performed, thereby obtaining voxels with a statistically significant different distribution of diffusion-related parameters including FA and linearity, planarity and sphericity coefficients, between controls and IUGR. The main goal of the use of VBA in this study was to explore and suggest potential relationships on all possible structural changes underlying the functional impairments in our IUGR model. Consequently, we decided to set a threshold of p<0.01 and we deliberately decided not to perform multiple comparisons correction. In addition to the analysis of differences on DTI parameters between cases and controls, the Spearman correlation between diffusion parameters and functional outcomes at each voxel was also calculated to identify which regions were related to the changes observed in the neurobehavioral and cognitive evaluation.

Since VBA requires the definition of a reference brain, the results may be biased by this choice. In order to avoid this bias and to increase the reliability of the results obtained, the VBA procedure was repeated taking all the subjects as template, and only the regions where differences were consistently noted in all the templates were considered. In this way, the variability produced by the arbitrary choice of the reference template is discarded.

#### 2.3- Connectivity analysis

Connectivity analysis within specific brain areas involved in anxiety, attention and short-term memory were evaluated. We defined two main brain networks:

Anxiety and attention network. The selection of areas was based on previous evidence that regulation of attention and emotional reactivity depends on the correct interaction between brainstem, limbic and cortical systems [70], [71]. Within the limbic system, the amygdala and hippocampus were included because of their role in fear and anxiety [72]–[74]. In addition, several cortical areas (frontal, temporal, cingulate cortices) and deep grey nuclei (striatum and thalamus) were selected due to their relation with attention and emotion [27], [75]–[78]. Moreover, some of these brain areas have been identified as components of the Papez circuit which has been proposed to play a major role in emotion [79]. Given these evidence, we arbitrarily defined the “anxiety and attentional network” as all those WM fibers passing through the amygdala and the hippocampus formation, and which additionally passed through at least one of the following structures: striatum, thalamus, prefrontal cortex, temporal cortex or cingulate cortex.
Short-term memory network. Brain areas proposed to be involved in short-term memory were selected. Although the exact type of memory encoded remains under debate, there is universal agreement that the hippocampus [80], [81], and especially the hippocampal formation [82], [83], have important roles in declarative memory. In addition, memory based on olfactory recognition depends of the temporal lobe, mainly of the perirhinal cortex [84] and performance of the Object Recognition Task has been proposed to rely on the correct interaction within the perirhinal-hippocampal-medial prefrontal network [85]–[87]. Finally, recent evidence has suggested the involvement of the thalamus in the regulation of short-term memory [88]. Based on these data, we arbitrarily defined the “short-term memory network” as all those WM fibers passing through the hippocampal formation and which additionally passed through at least one of the following structures: hippocampus, thalamus, prefrontal or temporal cortices.

Manual delineation of GM structures was performed in T1 weighted images including multiple cortical areas (prefrontal, cingulate, temporal), putamen, caudate nucleus, thalamus, amygdala, hippocampus and hippocampal formation (Figure 1, PANEL 4). Combining these regions with previously calculated tractography, WM fiber tracts involved in the two networks of short-term memory and anxiety were extracted. The measurement of connectivity within each network was assessed applying two different quantitative tractography metrics: 1) number of fibers within proposed networks corrected by the total number of fibers in each brain and 2) measurement of mean FA in fibers involved in the proposed networks. For both networks, we analyzed global circuit connectivity considering both right and left hemisphere fibers together (bilateral analysis), and specific right and left circuit connectivity, considering each hemisphere separately (right and left analysis). In addition, correlation between ratio of fibers and mean FA with functional test scores was also analyzed adjusting for gender.

### 3- Statistical analysis

For quantitative variables, normality was assessed by Shapiro-Wilk [89]. Results were expressed as mean and standard deviation (SD); whereas median and interquartile range (IQR) was used in non-normal variables. Differences between cases and controls were studied after adjusting for gender by means of general lineal model. In non-normal variables such analysis were performed after a log-transformation. For categorical variables, chi-squared test was used. SPSS 19.0 (SPSS Inc., Chicago, IL, USA) was used for this statistical analysis. In VBA approach, registered and smoothed volumes of FA, linearity, planarity and sphericity coefficients were used to obtain volumetric maps of t-statistics, showing the voxels that presented a significant difference between groups (uncorrected p<0.01). In addition, a correlation volume (ρ) was also calculated for each functional item, expressing positive and negative Spearman correlations between FA, linearity, planarity and sphericity coefficients and neurobehavioral and cognitive outcomes. VBA differences between groups and also in functional correlations were analyzed adjusting for gender. Image analysis, processing and regression analysis was performed by means of in-house software implemented in Matlab 2011a (The MathworksInc, Natick, MA, USA).

## Results

### 1- Sample characteristics

There were no surgical or postoperative complications in the 10 dams included. A total of 69 fetuses were included at the time of the induction (23 controls and 47 cases), 49 of which were alive at delivery (19 controls and 30 cases). Postnatally, 5 controls and 17 cases died within the first week of life, thus, 14 controls and 13 cases reached the long-term period. Overall, both the fetal and neonatal mortality rate was higher in cases (stillbirth 17.4% vs. 36.2%, p = 0.08 and neonatal mortality 26.3% vs. 56.7%, p = 0.01, controls vs. cases respectively). The birth weight was significantly lower in cases compared to controls (49.54 g (SD 5.85) vs. 38.34 g (SD 5.36), p≤0.001). Nevertheless, these differences were not observed at the 70th postnatal day (2747 g (SD 190) vs. 2626 g (SD 489), p = 0.41). Neither were any differences found in the time of postnatal evaluation (71 (IQR 3) vs. 70 (IQR 4) postnatal days, p = 0.099) nor in gender distribution (63.6% vs. 50% females, p = 0.425).

Of the 27 animals that were functionally evaluated, 6 animals (3 controls and 3 cases) were excluded from the final analysis due to gross tissue abnormalities resulting from sample extraction or manipulation observed in the standard MRI acquisition, with 21 animals in the final sample (11 controls and 10 cases). During the postnatal period, no gross motor abnormalities such as paresia or spasticity were observed in either group.

### 2- Neurobehavioral and cognitive outcomes: functional tests results

Concordance between the two functional tests from each blinded observer was explored using the interclass correlation coefficient which demonstrated good reliability (mean: 0.941).

In the Open Field Test, IUGR rabbits presented reduced exploratory activities, with a significantly increased latency of leaving the starting point and a trend to present reduced speed while exploring and less rearing. In addition, cases showed a significant reduction in time spent in the internal area as well as a reduction in the number of areas crossed in both the internal and external areas (Table 1).

**Table 1:** Open field behavioral results in study groups adjusted by gender.

	*Controls n = 11*	*Cases n = 10*	*p*
Latency of leaving the starting point, seconds †	3.0 (29.0)	59.0 (217.5)	*0.036*
Total squares crossed, number †	113.0 (26.0)	74.5 (54.0)	*0.272*
Total time exploring, seconds	424.5 (106.3)	330.1 (149.5)	*0.139*
Velocity of travelling (total squares/total time)	0.3 (0.1)	0.2 (0.1)	*0.395*
External squares crossed, number	101.2 (37.3)	65.2 (35.4)	*0.034*
Time in external squares, seconds †	578.0 (16.0)	598.0 (8.0)	*0.017*
Internal squares crossed, number	9.2 (4.5)	3.3 (3.2)	*0.004*
Time in internal squares, seconds †	22.0 (16.0)	2.0 (8.0)	*0.083*
Grooming, number †	1.0 (2.0)	0.0 (0.0)	*0.268*
Rearing, number	23.3 (10.3)	15.7 (11.2)	*0.165*

Results are mean and standard deviation (mean (SD)) in normal variables, with median and interquartile range (median (IQR)) in non-normal variables †.

**Figure 2:**
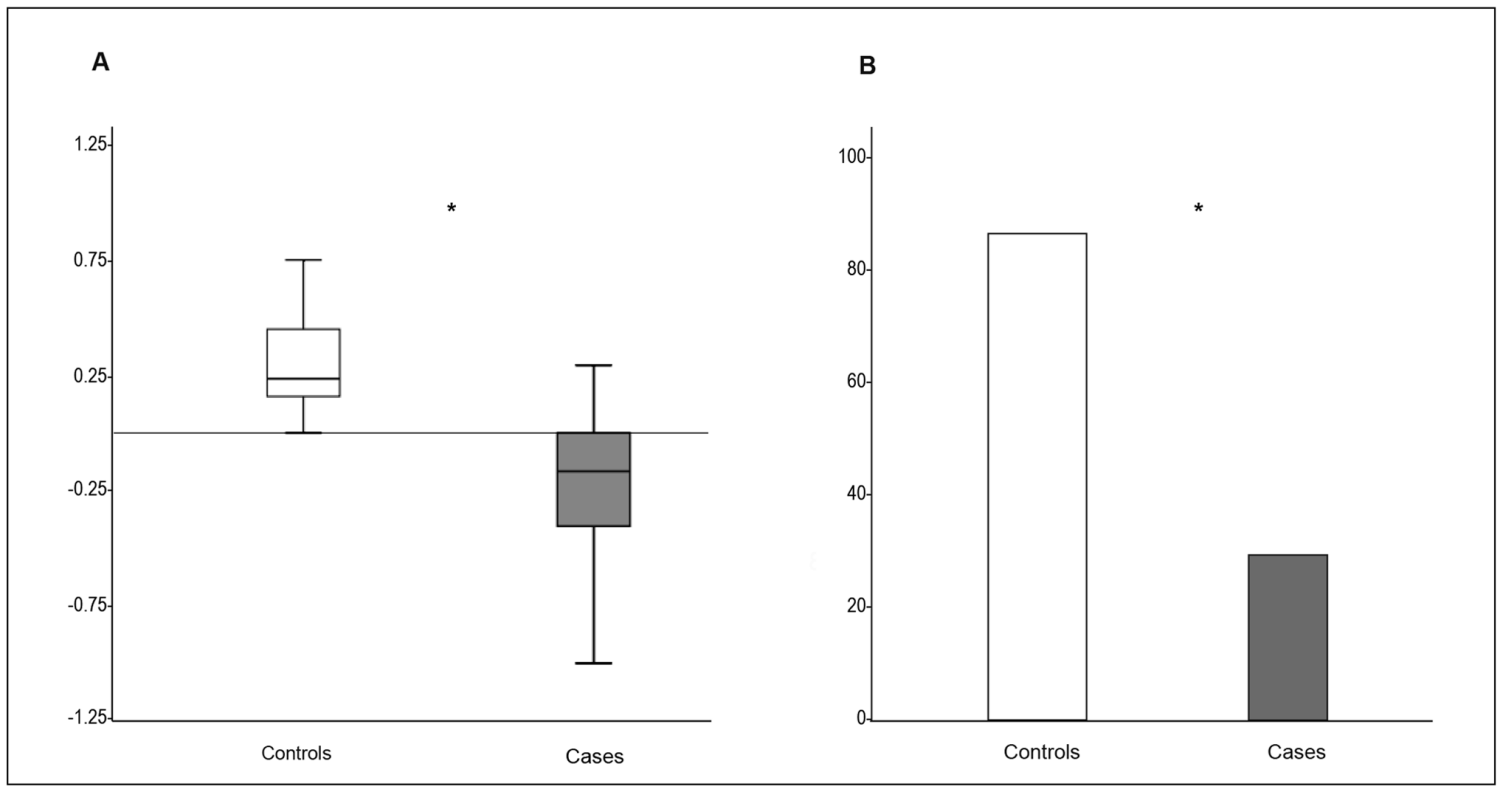
Discriminatory index results and percentage of learning in study groups. (A) Discriminatory index values of the study group (p = 0.013, adjusted for gender); (B) Percentage of controls and cases that reached the learning criteria (p = 0.03, adjusted for gender).

Regarding the Object Recognition Task, 7 cases and 8 controls fulfilled the previously established criteria. No differences were found in the time exploring right and left objects between groups in the Familiarization phase (right object: 9.50 s (SD 5.31) vs. 7.85 s (SD 0.04), p = 0.585; left object: 6.00 s (IQR 6.75) vs, 2.00 s (IQR 11.00), p = 0.69, controls vs. cases respectively). On the contrary, in the Testing phase controls spent significantly less time exploring the familiar object compared to cases (3.63 s (SD 1.92) vs. 6.71 s (SD 1.80), p =  0.011, controls vs. Cases, respectively). Interestingly, significantly decreased DI was observed in cases as well as a decreased proportion of rabbits achieving learning criteria (Figure 2).

Additionally we explored the relationship between birth weight and the neurobehavioral and cognitive measures, and as expected, we observed a significant correlation in almost all the parameters (Table S1).

### 3- MRI analysis

#### 3.1- Regional analysis: Voxel-based analysis

**Figure 3:**
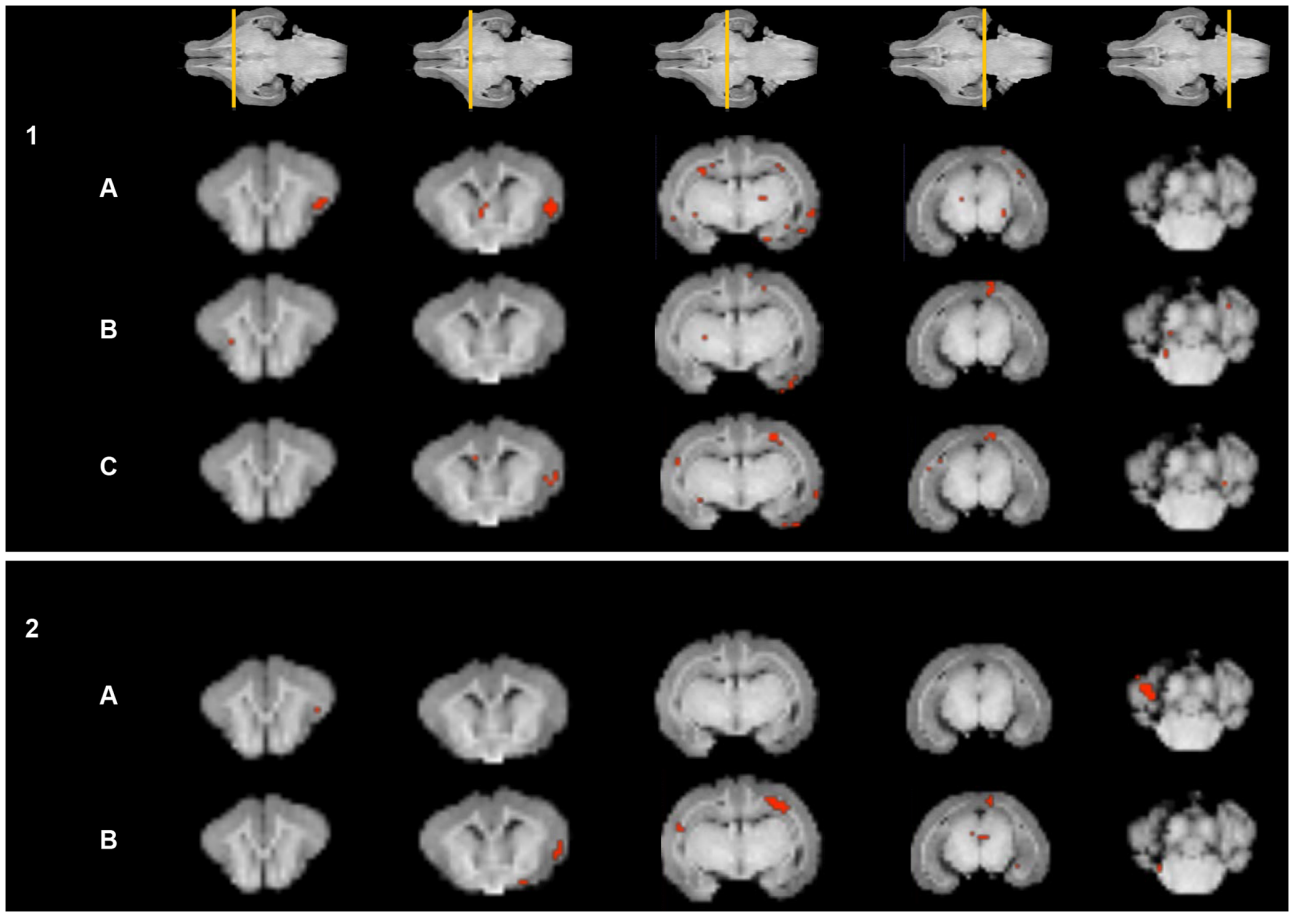
Fractional anisotropy, linearity, planarity and sphericity coefficients: regions showing statistically significant differences (p<0.01) between cases and controls. Coronal slices of the 3D reference image displaying representative anatomical structures for specific coefficients. Slice locations are shown in the T1 weighted images at the top. **PANEL 1:** Representative anatomical regions showing a significant decrease in linearity (A) and planarity (B) coefficients and in fractional anisotropy (C) in cases compared to controls. **PANEL 2:** Representative anatomical regions showing a significant increase in planarity (A) and sphericity (B) coefficients in cases compared to controls.

When VBA analysis was applied, statistically significant differences were found in FA distribution with a decreased FA in cases compared to controls in multiple structures including cortical regions (insular and temporal) and subventricular WM. The coefficient of linearity was also lower in cases in multiple areas including cortical regions (insular, temporal, prefrontal, and occipital), thalamus, superior colliculus, hippocampal formation and fimbria of hippocampus. The coefficient of planarity showed increased values in the occipital cortex and thalamus in IUGR rabbits, but decreased values in the insular cortex and cerebellar hemispheres. Finally, an increased coefficient of sphericity was observed in insular cortex and subventricular WM (Figure 3).

#### 3.2- Correlation between MRI diffusion and neurobehavioral and cognitive outcomes

**Figure 4:**
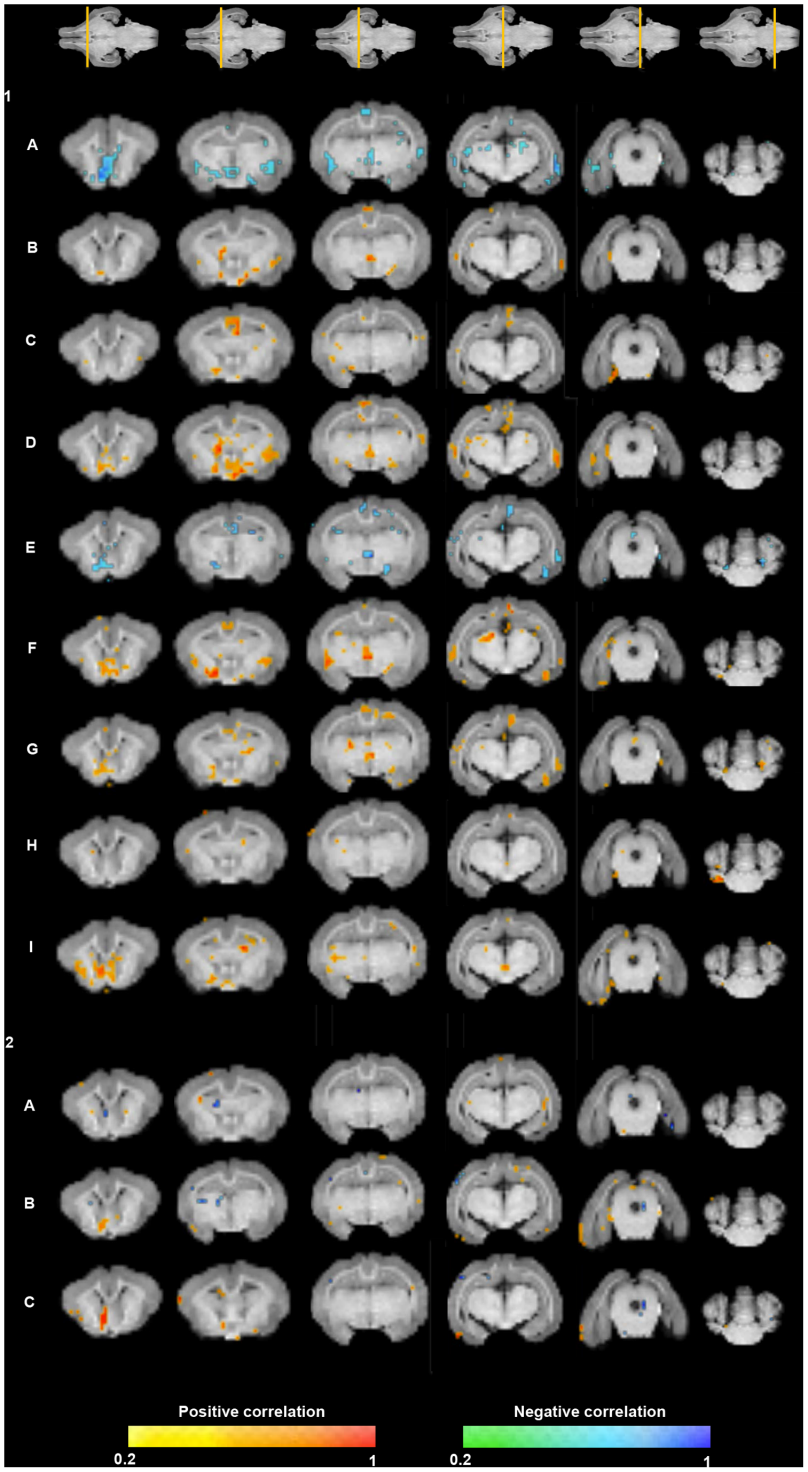
Correlation maps between neurobehavioral and cognitive tests items and fractional anisotropy values. Coronal slices (from anterior to posterior) of the 3D reference image are displayed. Colormap highlights the areas where the correlation coefficient is higher than 0.2. Spearman correlation p<0.001. **PLANEL 1:** (A) Latency of leaving the starting point, (B) Total squares crossed, (C) Total time exploring, (D) External squares crossed, (E) Time in external area, (F) Internal squares crossed, (G) Time in internal area, (H) Grooming, (I) Rearing. **PLANEL 2:** (A) Time exploring familiar object, (B) Time exploring novel object and (C) Discriminatory index.

The FA map shows correlations between functional variables, especially for the Open Field Test, and multiple brain areas (Figure 4 and Table 2). Regarding the GM structures, FA changes in hippocampus and hippocampal formation and in the cingulate and temporal cortex were correlated with more neurobehavioral domains; followed by the prefrontal cortex, thalamus and putamen nucleus. Interestingly, the amygdala presented a significant correlation with two of the variables that are strongly related to anxiety (number of squares crossed and time spent in the internal area). Within the WM structures, the anterior commissure and corona radiata areas showed more correlations with neurobehavioral and cognitive domains. All these findings were supported by similar changes in linearity, sphericity and planarity coefficients (Figure S1, Figure S2 and Figure S3).

**Table 2:** Significant correlations (p<0.01) between neurobehavioral and cognitive tests items and fractional anisotropy in brain regions adjusted by gender.

	*Positive correlation*	*Negative correlation*
**Open Field Behavioral Test**		
A		Temporal and cingulate cortices, putamen, thalamus, claustrum, anterior commissure
B	Cingulate cortex, claustrum, hippocampus, corpus callosum, anterior commissure, lateral lemniscus	
C	Cingulate, prefrontal and occipital cortices, hippocampal formation, corona radiata	
D	Prefrontal, temporal, cingulate and insular cortices, putamen, thalamus, claustrum, lateral lemniscus	
E		Cingulate cortex and hippocampus
F	Cingulate cortex, thalamus, amygdala, hippocampus, claustrum, superior colliculus, lateral lemniscus	
G	Cingulate and occipital cortices, amygdala, anterior comissure	
H	Caudate nucleus, cerebellar hemisphere	
I	Temporal cortex, hippocampus, thalamus, claustrum	
**Object Recognition Task**		
A	Occipital cortex, corona radiata	
B	Occipital cortex, anterior comissure	
C	Cingulate cortex, anterior comissure	

Open Field Behavioral Test items:(A) Latency of leaving the starting point, (B) Total squares crossed, (C) Total time exploring, (D) External squares crossed, (E) Time in external area, (F) Internal squares crossed, (G) Time in internal area, (H) Grooming, (I) Rearing; Object Recognition Task items: (A) Time exploring familiar object, (B) Time exploring novel object, and (C) Discriminatory index.

#### 3.3- Connectivity analysis

**Figure 5:**
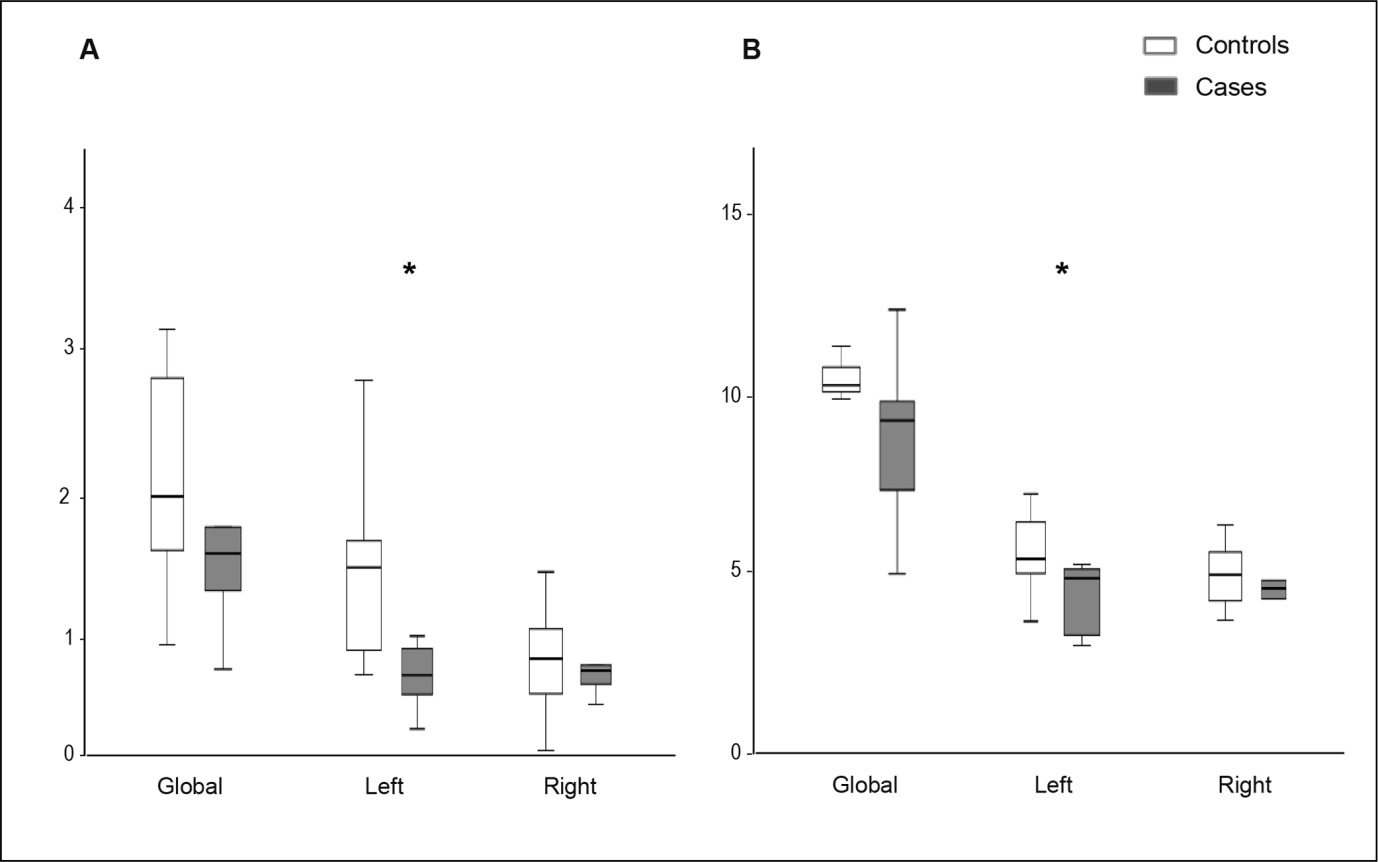
Ratio of fibers involved in the anxiety or memory networks over the total number of fibers. (A) Ratio of fibers adjusted for gender in anxiety network; in global analysis (p = 0.10), in left (p = 0.01) and right hemisphere (p = 0.59 *†*); (B) Ratio of fibers adjusted for gender in memory network; in global analysis (p = 0.08), in left (p = 0.03 *†*) and right hemisphere (p = 0.92 *†*). *†* Non-normal variables.

**Figure 6:**
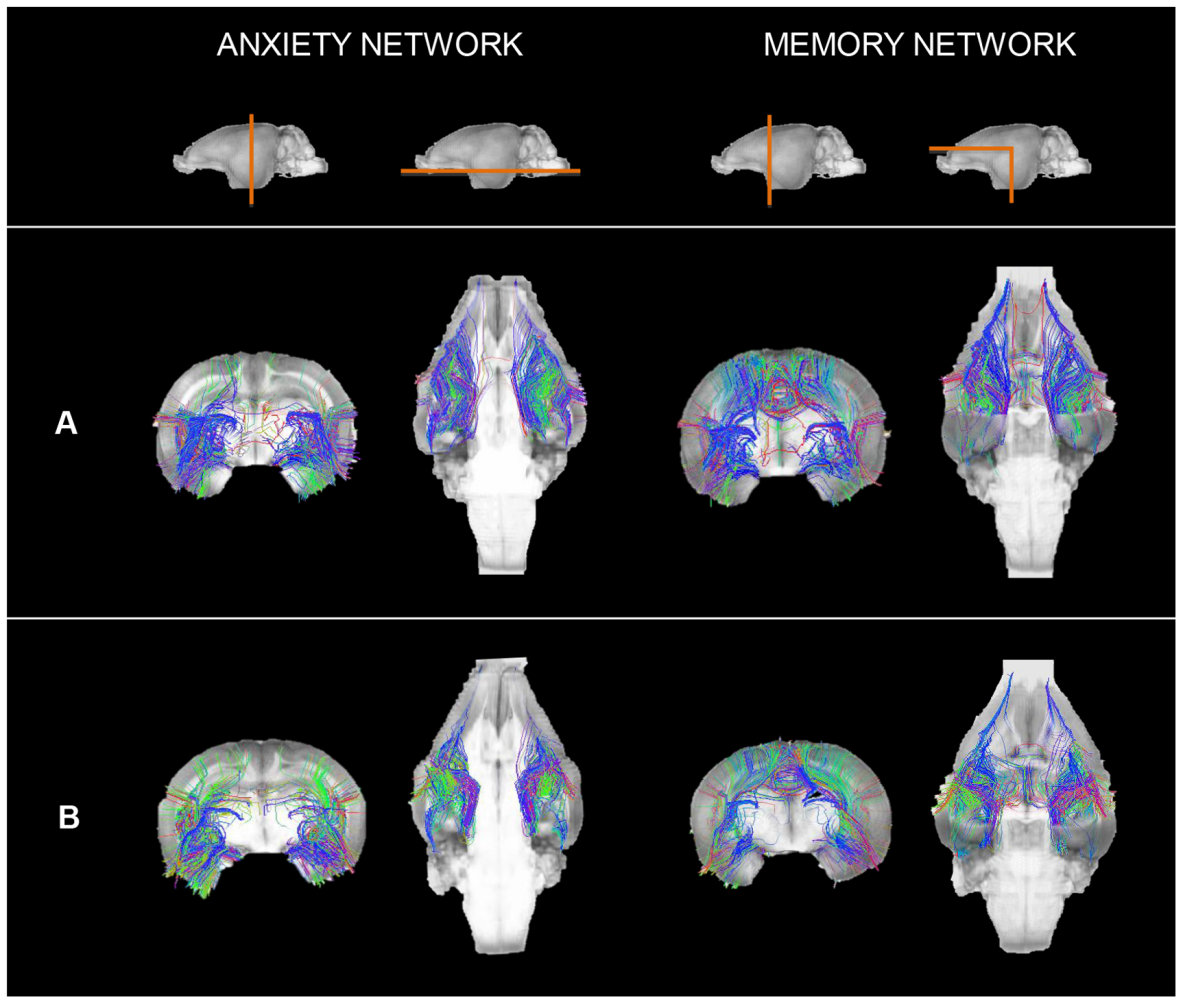
Reconstructed fibers in anxiety and memory networks in the experimental groups. Coronal and axial views of anxiety and memory networks of one control (A) and one case (B). Reconstructed fibers are overlapped to the 3D reconstruction of T1 weighted images.

Analysis of the total number of WM fiber tracts reconstructed for the whole brain did not differ between groups (14775 (SD 2332) vs. 13921 (SD 2148), p = 0.371, controls vs. cases). Nevertheless, on evaluation of the percentage of fibers involved in a specific network, the cases showed a trend to present a lower ratio of fibers in both networks; being statistically significant in the left hemisphere for both networks (Figure 5 and 6). Table 3 depicts the mean correlation coefficients between the percentage of fibers and functional test results. Regarding the anxiety network, the left hemisphere was significantly correlated with nearly all the variables in the Open Field Test, whereas in the memory network any variable achieved statistical significance. Finally, we did not observe significant differences in the mean FA in the two networks, although there was a trend to presenting a lower FA in cases compared to controls, especially in the anxiety network (Table 4). In addition, we did not observe a correlation between FA results and the functional results (data not shown).

**Table 3:** Mean correlation coefficients between ratio of fibers in anxiety and short-term memory networks and neurobehavioral and cognitive tests item results (Spearman's correlation) adjusted by gender.

	*Global*	*Left Hemisphere*	*Right hemisphere*
**Anxiety network**			
A	−0.30	**−0.50** *	−0.06
B	0.30	**0.56** **	−0.05
C	−0.01	0.13	−0.01
D	0.30	**0.55** *	−0.05
E	−0.43	**−0.49** *	−0.10
F	0.35	**0.52** *	0.10
G	0.43	**0.48** *	0.10
H	0.05	−0.05	0.24
I	0.18	0.18	0.22
**Memory network**			
A	−0.51	−0.31	−0.42
B	0.18	0.34	−0.45
C	0.50	0.46	0.11

Open Field Behavioral Test items: (A) Latency of leaving the starting point, (B) Total squares crossed, (C) Total time exploring, (D) External squares crossed, (E) Time in external area, (F) Internal squares crossed, (G) Time in internal area, (H) Grooming, (I) Rearing. Object Recognition Task items: (A) Time exploring familiar object, (B) Time exploring novel object, and (C) Discriminatory index.

* <p 0.05.

** <p 0.01.

**Table 4:** Fractional anisotropy values of fiber tracts within each network adjusted by gender.

	*Controls n = 11*	*Cases n = 10*	*p*
**Anxiety network**			
Global	0.2539 (0.0161)	0.2448 (0.0250)	*0.319*
Left	0.2512 (0.0164)	0.2478 (0.0332)	*0.759*
Right	0.2519 (0.0408)	0.2391 (0.0283)	*0.367*
**Memory network**			
Global †	0.2310 (0.0112)	0.2197 (0.0181)	*0.128*
Left	0.2322 (0.0118)	0.2203 (0.0216)	*0.162*
Right †	0.2233 (0.0273)	0.2219 (0.0128)	*0.128*

Results are mean and standard deviation (mean (SD)) in normal variables, with median and interquartile range (median (IQR)) in non-normal variables †.

Again, we explored the relationship between birth weight and the ratio of fibers in both networks observing significant correlations in almost all ratios for both networks (Table S2).

## Discussion

To the best of our knowledge, this is the first report simultaneously characterizing long-term cognitive neurobehavioral and cognitive dysfunctions and the related neuroanatomical changes in a near-term IUGR rabbit model using advanced imaging techniques. It has been described that neurobehavioral and cognitive impairments associated with IUGR mainly comprise short-term memory, attention and anxiety, but neuroanatomical correlations have not as yet been reported.

### 1- Long-term neurobehavior and cognitive results

Results from the Open Field Test showed that IUGR rabbits presented a higher degree of anxiety expressed by reduced exploratory activities similar to what has been described in rat models after acute hypoxic-ischemic injury [90]. In addition, there was an increase in the time spent in the periphery and in the latency after leaving the starting point, a characteristic sign of anxiety in animals [91]. Likewise, the IUGR rabbits presented decreased grooming activity. Although the interpretation of grooming behavior in rodents is complex, changes in the incidence of this particular behavior have been also related to altered levels of anxiety [92]. Increased anxiety has been described in rats after perinatal hypoxic insult [93], and in human adolescents and adults with a history of IUGR [11], [24]. Data derived from the Object Recognition Task have demonstrated that the IUGR rabbit model demonstrates short-term memory and attentional disorders similar to what has been reported in humans [13]. Our results are comparable to those obtained in rats after prenatal unilateral uterine artery occlusion [94], [95].

Overall, with the application of these two tests we have demonstrated that the surgical model of IUGR in pregnant rabbits reproduces some of the cognitive and neuropsychological features described in IUGR children.

### 2- MRI regional analysis

Long-term structural changes were more remarkable in GM areas and included multiple cortical regions (insular, temporal, prefrontal, occipital cortices and cerebellar hemisphere) and deep GM nuclei (thalamus and hippocampus). Interestingly, our findings of DTI changes in the prefrontal and entorhinal cortices and hippocampus are in line with previous evidence aimed at describing histology changes in the long-term period in the offspring of pregnant rats with IUGR after prenatal occlusion of the unilateral uterine artery. These histological changes include a decreased number of neurons, astrogliosis, an increase in GABAergic neurons and diffuse axonal degeneration [94], [95]. Changes in GM detected by DTI have been proposed to reflect changes in the dendritic architecture of pyramidal cells [31], [96] which could, in turn, suggest a connectivity impairment of these GM structures. Concerning WM, regional analysis of DTI parameters revealed significant differences with decreased FA and linearity and increased sphericity values in the fimbria of the hippocampus and in the subventricular WM in IUGR group. FA values are closely related to myelination process, increasing its values in WM areas during brain maturation [31]. Decreased values of FA in WM tracts have previously been described after mild hypoxic-isquemic injury and correlated with decreased myelin content, persisting these changes after the recovery period [97]. Consistently with decreased FA, IUGR showed decreased linearity and increased sphericity coefficients that are related with less organized fiber tracts in WM bundles [63]. Therefore, our results support the hypothesis that IUGR is related with an altered and delayed WM organization and maturation that persists even at long-term period. It should be noted that WM changes seemed to be less pronounced in comparison with our previous findings in which structural brain changes in the neonatal period were assessed using the same animal model [34]. One explanation for the few differences observed in WM structures could be derived for the voxel size used. It should be taken into account that a voxel size of 0.7×0.7×0.7 mm^3^ may produce some partial volume effects which may hinder the presence of differences in some small brain areas, such as thin WM tracts. If these partial volume effects had been present, they would have resulted in a conservative bias, thus attenuating the existing differences and not affecting the validity of the differences observed. Aside from methodological limitations, the assignment of most of the diffusion changes observed to GM compared to WM may indicate that long-term brain plasticity throughout childhood and adolescence [98], [99] is more efficient at correcting WM than GM deficits. In line with this notion, myelin content increases from the neonatal period up to young adulthood in an IUGR surgical guinea pig model [100]. The same histological findings, together with a reduction in the magnitude of differences with respect to controls in FA, have been reported in long-term as compared with neonatal measurements in rats [97]. Regional changes in FA showed significant correlations mostly in GM structures with functional results, especially those related to the Open Field Test. With this test, the hippocampal complex, prefrontal and cingulate cortices presented the highest number of correlations. Animal studies have demonstrated the important role of a normal functioning of the hippocampus in the regulation of anxiety [73], [101], [102]. Concerning prefrontal and cingulate cortices, reduced volumes in children with ADHD [103] and healthy individuals [104] as well as histological changes in rodents [105] in these structures have been associated with attention and anxiety traits. In addition, changes in diffusion MRI parameters of the amygdala were correlated with the number of squares crossed and the time spent in the internal area, two items strongly related to anxiety. These findings are in line with the reported role of the amygdala in the processing of fear and anxiety [72], [73].

Concerning correlations with the Object Recognition Task, within GM structures we observed a significant correlation between regional FA changes in the cingulate cortex and the Object Recognition Task results. Several experimental studies in rodents have found that the cingulate cortex plays a key role in novelty detection, attention and memory in fearful situations [106]–[109], and any disruption in this structure could impair memory consolidation [110]. Taking this into account, although the Object Recognition Task was conducted in the same arena in which the rabbit had previously performed the Open Field Test, persistence of any degree of anxiety while performing the Object Recognition Task could not be ruled out. This could impair memory consolidation in those animals with structural changes in the cingulate, such as our results suggest. This suggestion is in line with clinical studies that have postulated that short-term memory problems observed in IUGR children may be accounted for by a lack of sufficient attention rather than a deficit in processing the information per se [13], impeding short-term memory function. Regarding WM and Object Recognition Task results, the most consistent correlations, as they were observed in all the DTI parameters, were found in the anterior commissure and corona radiata. These tracts connect several brain areas that are engaged in memory and attention [111]–[113]. Contrary to our original hypotheses, we did not observe any significant correlations between GM and Object Recognition Task results in brain areas classically described to be involved in memory recognition, such as hippocampal formation, temporal lobe and prefrontal cortex [80]–[82], [84],[86]. These findings suggest that short-term memory impairment induced by IUGR as reflected in the Object Recognition Task could depend more on the connectivity between relevant regions than on intrinsic changes in their GM. This notion is in line with previous findings supporting strong dependence of memory formation and on the integrity of the perirhinal-hippocampal-medial prefrontal network [85]–[87].

In summary, these results partially confirm the hypotheses formulated in clinical studies on children and adolescents with IUGR, but provide new insight as to the specific structural anomalies underlying neurobehavioral and cognitive impairments.

### 3- Connectivity analysis

IUGR showed a decreased number of fibers in anxiety, attention and memory networks over the total number of fibers reconstructed. These differences were statistically significant in the left hemisphere, with a trend to decreased FA in both networks. Moreover, a significant correlation was observed between the ratio of fiber in the left hemisphere for anxiety network and functional results. Overall, our results are in line with previous MRI diffusion studies in patients with anxiety and attention disorders or memory impairments. Changes in connectivity within the prefrontal and anterior cingulate cortexes and the amygdala have been correlated with anxiety [78], [114], [115]. In addition, microstructural changes in the connectivity within the fronto-striatal pathway and WM tracts connecting the amygdala and the prefrontal cortex have been described to be strongly related to the ADHD disorder in children and adolescents [37], [116]–[118]. Concerning the memory network, reduced FA has been observed in WM tracts connecting the temporal cortex and the hippocampus in children [119] and in the corona radiata in adults with mild traumatic injury [112]. In these studies, decreased FA was correlated with object recognition results in children, and with attentional and memory impairment in adults. In addition, changes in parahippocampal WM that connects the entorhinal cortex with the hippocampus were correlated with declarative memory problems in elderly patients with mild cognitive impairment [120], [121]. Most of the differences observed in our study were restricted to the left hemisphere. Differences observed in the left anxiety network are consistent with previous evidence suggesting left hemisphere lateralization in fear-related anxiety processing [122].

Altogether, the results of this study support the contention that altered connectivity patterns within regions involved in anxiety, attention and memory are involved in the functional impairment associated with IUGR that persists up to the preadolescent period and suggests the importance of completing the normal programming of neuronal connectivity patterns for the achievement of normal neurodevelopment. The data reported demonstrate a decreased number of fibers in combination with more modest changes in FA. These results are different to those observed in the neonatal period [34], and support the idea that, in the long-term, structural changes are essentially related to the distribution rather than with the integrity of fibers. These findings are in line with previous evidence demonstrating that delayed myelination during critical developmental periods can be restored later [100], but will lead to long-term consequences in the patterns of connectivity, as has been consistently demonstrated in human and experimental studies [123], [124].

### 4- Methodological considerations and limitations of the study

The methodology used to perform both VBA and connectivity analysis in this study deserves some discussion. With regard to connectivity analysis, we acknowledge that the networks defined in this study have not been fully validated, although we used consistent evidence from the literature demonstrating the involvement of all the selected regions in the functions of interest. In addition, we acknowledge that there are no standard or widely validated approaches for quantifying tractography metrics in defined networks. Several studies have previously used this approach in human studies to characterize changes in brain structure and its neurobehavioral correlates in neurodevelopmental diseases such as ADHD [37], focal perinatal brain injury [125], and periventricular leucomalacia [39], [40]. Only a few studies have used fiber count to assess the connectivity within specific brain areas [39], [40], [126] and these studies did not adjust for brain size or total number of fibers reconstructed. In the present study we introduced this methodological change in order to counter the potential bias of differences in the total number of fibers reconstructed from case to case. Regarding VBA, the use of this approach implies weaker statistical power due to the large number of voxels tested [67], increasing type I error rate. This is partially compensated by the smoothing after registering the DTI volumes to the reference. By smoothing the DTI maps, the effective number of multiple comparisons in the statistical testing was reduced, thereby improving statistical power [67]. We acknowledge that not correcting for multiple comparisons introduces a bias in the interpretation of results. However, as noted above, we intended to use this method in an exploratory fashion which allowed to suggest potential relationships. We would like to stress that confirmation of the relationships here suggested requires further studies with larger sample sizes. Another issue concerning VBA is that the method requires registration of all the subjects in the dataset to a template volume, and therefore the arbitrary choice of this template could bias the result [67]. As described in the methodology section, this issue was addressed by repeating the VBA considering each of the subjects as the reference. Finally, we did not include ADC data in the regional analysis since the fixation process decreases the water content in brain tissue, reducing absolute ADC values in a non-homogeneous and, therefore, non-predictable manner [127], especially in hypoxic tissue [128].

From the point of view of the experimental design, the high mortality rate during the first postnatal week may have selected less severe cases for the long-term follow up, thus attenuating the true impact of the condition. Despite this conservative bias, we were able to demonstrate structural and functional changes after IUGR. Finally, we acknowledge that our sample size may be underpowered to evaluate gender differences in the variables assessed. However, we decided to include gender as a potential confounder in our analysis since adjustment is recommended when biologically confounding is likely, as occurs in many neurobehavioral processes [129].

## Conclusions

In conclusion, we have developed a rabbit model reproducing functional and neurostructural consequences of near-term IUGR which persist up to young adulthood. Diffusion MRI demonstrated differences in the specific brain regions involved in the regulation of anxiety, attention and memory and in their related networks which were correlated with long-term functional impairments. The study provides evidence of the type of structural changes involved in long-term neurodevelopmental anomalies associated with IUGR and support the potential value of methods based on diffusion quantitative metrics to assess changes associated with brain reorganization that are not demonstrable by standard imaging techniques. Using the methodology described herein, further multi-scale studies could be performed in order to advance the understanding of the prenatal mechanisms underlying abnormal neurodevelopment to thereby target potential biomarkers based on diffusion MRI and connectivity analysis for early diagnosis and monitoring of the impact of interventional studies.

## Supporting Information

Figure S1 **Correlation maps between neurobehavioral and cognitive tests items and linearity coefficient.** Coronal slices (from anterior to posterior) of the 3D reference image are displayed. Colormap highlights the areas where each correlation coefficient is higher than 0.2. Spearman correlation p<0.001. **PLANEL 1:** (A) Latency of leaving the starting point, (B) Total squares crossed, (C) Total time exploring, (D) External squares crossed, (E) Time in external area, (F) Internal squares crossed, (G) Time in internal area, (H) Grooming, and (I) Rearing. **PLANEL 2:** (A) Time exploring familiar object, (B) Time exploring novel object, and (C) Discriminatory index.
(TIF)

Figure S2 **Correlation maps between neurobehavioral and cognitive tests items and sphericity coefficient.** Coronal slices (from anterior to posterior) of the 3D reference image are displayed. Colormap highlights the areas where each correlation coefficient is higher than 0.2. Spearman correlation p<0.001. **PLANEL 1:** (A) Latency of leaving the starting point, (B) Total squares crossed, (C) Total time exploring, (D) External squares crossed, (E) Time in external area, (F) Internal squares crossed, (G) Time in internal area, (H) Grooming, and (I) Rearing. **PLANEL 2:** (A) Time exploring familiar object, (B) Time exploring novel object, and (C) Discriminatory index.
(TIF)

Figure S3 **Correlation maps between neurobehavioral and cognitive tests items and planarity coefficient.** Coronal slices (from anterior to posterior) of the 3D reference image are displayed. Colormap highlights the areas where each correlation coefficient is higher than 0.2. Spearman correlation p<0.001. **PLANEL 1:** (A) Latency of leaving the starting point, (B) Total squares crossed, (C) Total time exploring, (D) External squares crossed, (E) Time in external area, (F) Internal squares crossed, (G) Time in internal area, (H) Grooming, and (I) Rearing. **PLANEL 2:** (A) Time exploring familiar object, (B) Time exploring novel object, and (C) Discriminatory index.
(TIF)

Table S1 **Mean correlation coefficients between functional results and birth weight (Spearman's correlation).**
(DOC)

Table S2 **Mean correlation coefficients between ratios of fibers and birth weight (Spearman's correlation).**
(DOC)
