# Supplementary figures and images for: Long-Term Functional Outcomes and Correlation with Regional Brain Connectivity by MRI Diffusion Tractography Metrics in a Near-Term Rabbit Model of Intrauterine Growth Restriction
Source: PLoS One. 2013 Oct 15;8(10):e76453. doi: 10.1371/journal.pone.0076453 (PMC3797044; doi:10.1371/journal.pone.0076453)

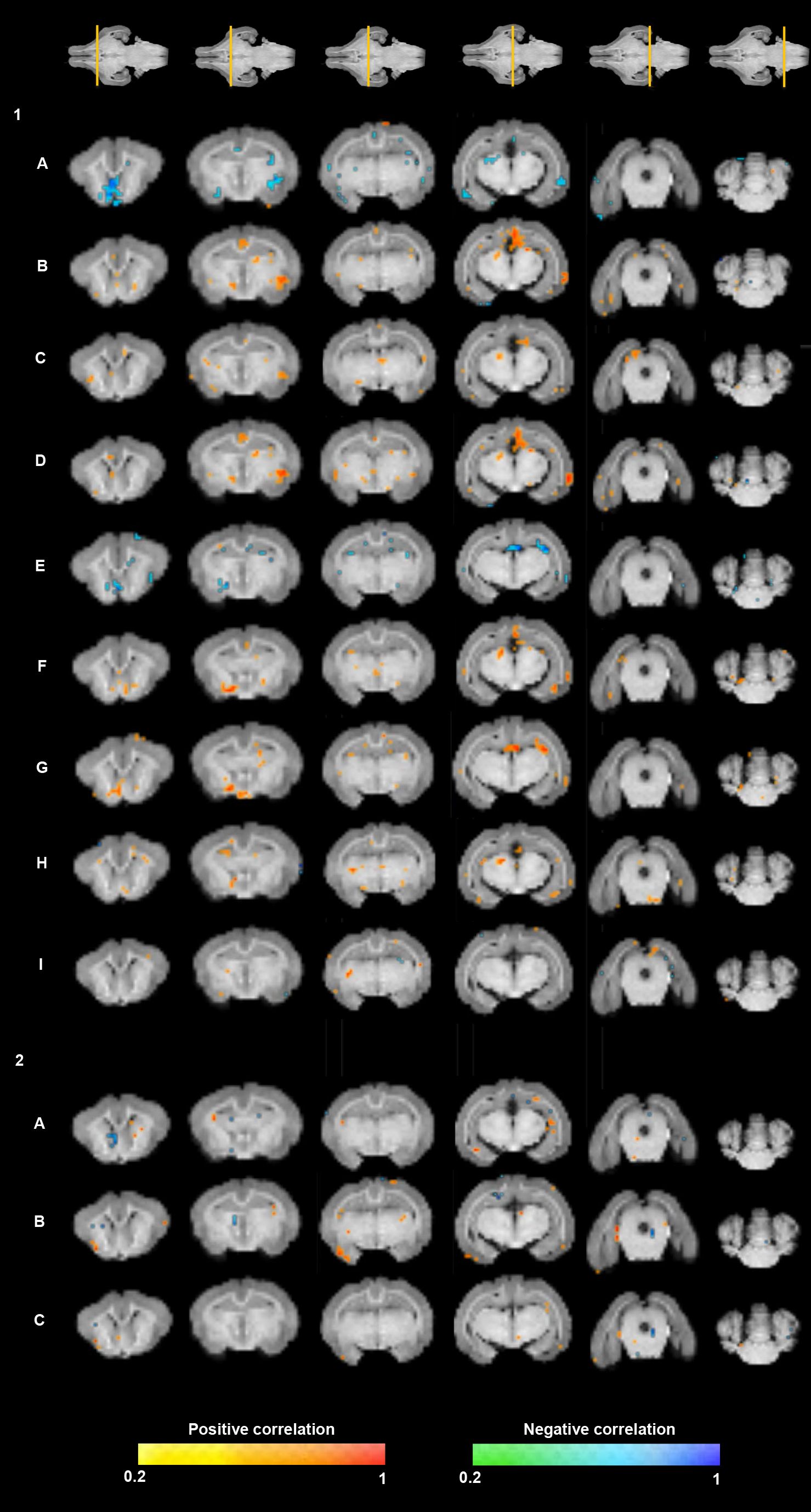

Supplement: Figure S1 — Correlation maps between neurobehavioral and cognitive tests items and linearity coefficient. Coronal slices (from anterior to posterior) of the 3D reference image are displayed. Colormap highlights the areas where each correlation coefficient is higher than 0.2. Spearman correlation p<0.001. PLANEL 1: (A) Latency of leaving the starting point, (B) Total squares crossed, (C) Total time exploring, (D) External squares crossed, (E) Time in external area, (F) Internal squares crossed, (G) Time in internal area, (H) Grooming, and (I) Rearing. PLANEL 2: (A) Time exploring familiar object, (B) Time exploring novel object, and (C) Discriminatory index. (TIF) [file pone.0076453.s001.tif]

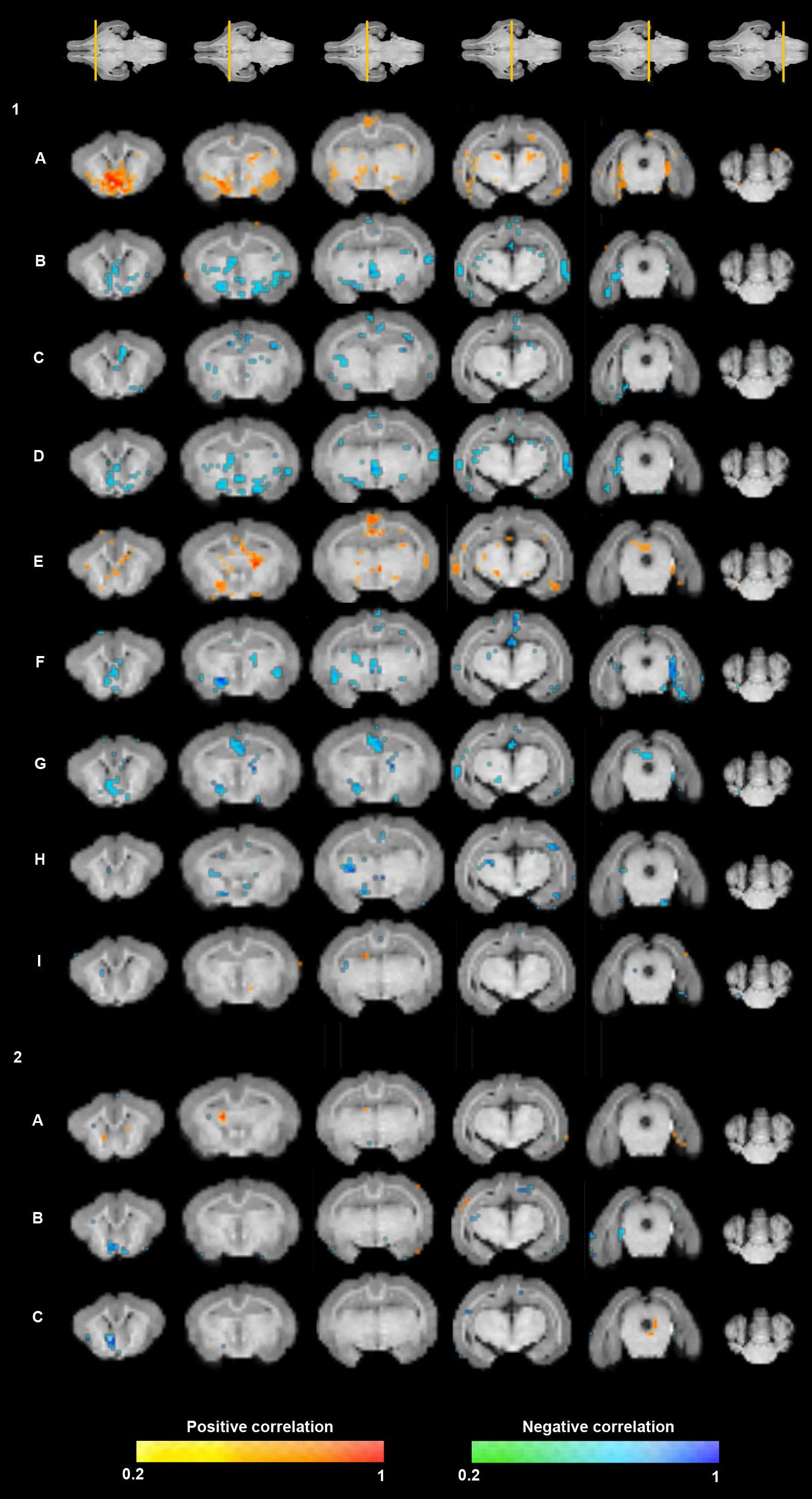

Supplement: Figure S2 — Correlation maps between neurobehavioral and cognitive tests items and sphericity coefficient. Coronal slices (from anterior to posterior) of the 3D reference image are displayed. Colormap highlights the areas where each correlation coefficient is higher than 0.2. Spearman correlation p<0.001. PLANEL 1: (A) Latency of leaving the starting point, (B) Total squares crossed, (C) Total time exploring, (D) External squares crossed, (E) Time in external area, (F) Internal squares crossed, (G) Time in internal area, (H) Grooming, and (I) Rearing. PLANEL 2: (A) Time exploring familiar object, (B) Time exploring novel object, and (C) Discriminatory index. (TIF) [file pone.0076453.s002.tif]

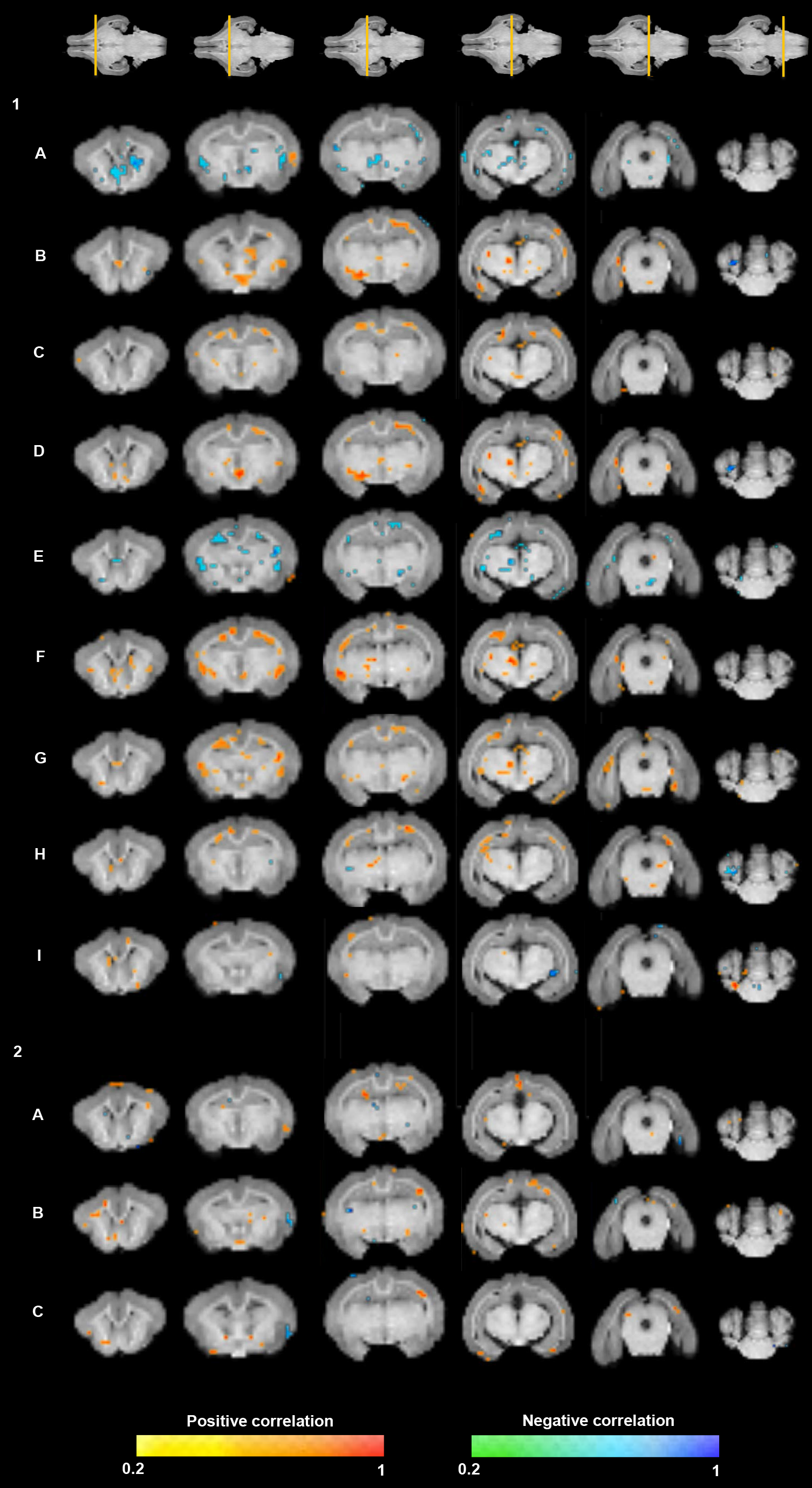

Supplement: Figure S3 — Correlation maps between neurobehavioral and cognitive tests items and planarity coefficient. Coronal slices (from anterior to posterior) of the 3D reference image are displayed. Colormap highlights the areas where each correlation coefficient is higher than 0.2. Spearman correlation p<0.001. PLANEL 1: (A) Latency of leaving the starting point, (B) Total squares crossed, (C) Total time exploring, (D) External squares crossed, (E) Time in external area, (F) Internal squares crossed, (G) Time in internal area, (H) Grooming, and (I) Rearing. PLANEL 2: (A) Time exploring familiar object, (B) Time exploring novel object, and (C) Discriminatory index. (TIF) [file pone.0076453.s003.tif]
