# Supplementary material for: Long-Term Functional Outcomes and Correlation with Regional Brain Connectivity by MRI Diffusion Tractography Metrics in a Near-Term Rabbit Model of Intrauterine Growth Restriction
Source: PLoS One. 2013 Oct 15;8(10):e76453. doi: 10.1371/journal.pone.0076453 (PMC3797044; doi:10.1371/journal.pone.0076453)
Supplement: Table S1 — Mean correlation coefficients between functional results and birth weight (Spearman's correlation). (DOC) [file pone.0076453.s004.doc]

**Table S1. Mean correlation coefficients between functional results and birth** weight (Spearman’s correlation).

|  | ***correlation coefficient (rho)*** | ***p*** |
| --- | --- | --- |
| **Open field behavioral test variables** | | |
| Latency of leaving the starting point, seconds | -0.52 | *0.02* |
| Total squares crossed, number | 0.48 | *0.03* |
| Total time exploring, seconds | 0.16 | *0.51* |
| External squares crossed, number | 0.49 | *0.03* |
| Time in external squares, seconds | -0.58 | *0.01* |
| Internal squares crossed, number | 0.40 | *0.08* |
| Time in internal squares, seconds | 0.53 | *0.02* |
| Grooming, number | 0.07 | *0.76* |
| Rearing, number | 0.20 | *0.41* |
| **Object recognition task variables** | | |
| Time exploring familiar object, seconds | -0.45 | *0.18* |
| Time exploring familiar object, seconds | 0.55 | *0.04* |
| Discriminatory index | 0.66 | *0.01* |
