# Supplementary material for: Long-Term Functional Outcomes and Correlation with Regional Brain Connectivity by MRI Diffusion Tractography Metrics in a Near-Term Rabbit Model of Intrauterine Growth Restriction
Source: PLoS One. 2013 Oct 15;8(10):e76453. doi: 10.1371/journal.pone.0076453 (PMC3797044; doi:10.1371/journal.pone.0076453)
Supplement: Table S2 — Mean correlation coefficients between ratios of fibers and birth weight (Spearman's correlation). (DOC) [file pone.0076453.s005.doc]

**Table S2. Mean correlation coefficients between ratios of fibers and birth weight (Spearman’s correlation).**

|  | ***correlation coefficient (rho)*** | ***p*** |
| --- | --- | --- |
| **Anxiety network** |  |  |
| Global ratio of fibers | 0.46 | *0.04* |
| Left hemisphere ratio of fibers | 0.12 | *0.62* |
| Right hemisphere ratio of fibers | 0.54 | *0.01* |
| **Memory network** |  |  |
| Global ratio of fibers | 0.68 | *0.00* |
| Left hemisphere ratio of fibers | 0.31 | *0.18* |
| Right hemisphere ratio of fibers | 0.42 | *0.06* |
